# Human Endogenous Retrovirus HERV-Fc1 Association with Multiple Sclerosis Susceptibility: A Meta-Analysis

**DOI:** 10.1371/journal.pone.0090182

**Published:** 2014-03-03

**Authors:** Belén de la Hera, Jezabel Varadé, Marta García-Montojo, Antonio Alcina, María Fedetz, Iraide Alloza, Ianire Astobiza, Laura Leyva, Oscar Fernández, Guillermo Izquierdo, Alfredo Antigüedad, Rafael Arroyo, Roberto Álvarez-Lafuente, Koen Vandenbroeck, Fuencisla Matesanz, Elena Urcelay

**Affiliations:** 1 Immunology Dept., Hospital Clínico San Carlos, Instituto de Investigación Sanitaria del Hospital Clínico San Carlos (IdISSC), Madrid, Spain; 2 Multiple Sclerosis Unit, Neurology Dept., Hospital Clínico San Carlos, Instituto de Investigación Sanitaria del Hospital Clínico San Carlos (IdISSC), Madrid, Spain; 3 Instituto de Parasitología y Biomedicina “López Neyra”, Consejo Superior de Investigaciones Científicas (CSIC), Granada, Spain; 4 Neurogenomiks Group, Universidad del País Vasco (UPV/EHU), Leioa, Spain; 5 Laboratorio de Investigación, Instituto de Neurociencias Clínicas, Hospital Regional Universitario, Málaga, Spain; 6 Servicio de Neurología, Instituto de Neurociencias Clínicas, Hospital Regional Universitario, Málaga, Spain; 7 Unidad de Esclerosis Múltiple, Hospital Virgen Macarena, Sevilla, Spain; 8 Servicio de Neurología, Hospital de Basurto, Bilbao, Spain; 9 Achucarro Basque Center for Neuroscience – UPV/EHU, Zamudio, Spain; 10 IKERBASQUE, Basque Foundation for Science, Bilbao, Spain; Hospital Nacional de Parapléjicos – SESCAM, Spain

## Abstract

**Background:**

Human endogenous retroviruses (HERVs) are repetitive sequences derived from ancestral germ-line infections by exogenous retroviruses and different HERV families have been integrated in the genome. HERV-Fc1 in chromosome X has been previously associated with multiple sclerosis (MS) in Northern European populations. Additionally, HERV-Fc1 RNA levels of expression have been found increased in plasma of MS patients with active disease. Considering the North-South latitude gradient in MS prevalence, we aimed to evaluate the role of HERV-Fc1on MS risk in three independent Spanish cohorts.

**Methods:**

A single nucleotide polymorphism near HERV-Fc1, rs391745, was genotyped by Taqman chemistry in a total of 2473 MS patients and 3031 ethnically matched controls, consecutively recruited from: Northern (569 patients and 980 controls), Central (883 patients and 692 controls) and Southern (1021 patients and 1359 controls) Spain. Our results were pooled in a meta-analysis with previously published data.

**Results:**

Significant associations of the HERV-Fc1 polymorphism with MS were observed in two Spanish cohorts and the combined meta-analysis with previous data yielded a significant association [rs391745 C-allele carriers: p_M-H_ = 0.0005; OR_M-H_ (95% CI) = 1.27 (1.11–1.45)]. Concordantly to previous findings, when the analysis was restricted to relapsing remitting and secondary progressive MS samples, a slight enhancement in the strength of the association was observed [p_M-H_ = 0.0003, OR_M-H_ (95% CI) = 1.32 (1.14–1.53)].

**Conclusion:**

Association of the HERV-Fc1 polymorphism rs391745 with bout-onset MS susceptibility was confirmed in Southern European cohorts.

## Introduction

Multiple sclerosis (MS) is a complex autoimmune disorder characterized by multifocal demyelination, axonal loss and neurodegeneration within the central nervous system of genetically susceptible individuals [1]. Clinical symptoms vary according to the location of the neurological lesions and patients often suffer an initial clinical isolated syndrome followed by a series of recurring-remitting events with neurological impairment (RRMS). Usually patients recover their near normal neurological function after each episode, but with the course of the disease an irreversible progression of clinical disability termed secondary progression (SPMS) may appear and early therapeutic intervention is claimed to delay this process. In only 10–15% of MS patients a clinical progression from the debut of the disease is observed, referred to as primary progressive MS (PPMS).

MS aetiology remains elusive, but the prevailing hypothesis supports an underlying autoimmune process triggered by the interplay of not fully described environmental and genetic factors. The weight of either genetics or environment in the pathogenesisof MS is a matter of debate [2]. In terms of environmental factors, higher latitude has been reported to correlate with increased prevalence of MS [3] probably driven by differences in sunlight exposure and vitamin D levels [4], [5], also repeatedly mentioned as environmental factors related with the risk of developing MS [6]. In addition, several viruses have been considered as triggers of MS, among them Epstein Barr virus [7], [8], Herpes Simplex or human herpesviruses [9], which could be key players in the disease mediated through direct or indirect mechanisms.

The strongest genetic factor to date, the Human Leucocyte Antigen (HLA) locuswas identified almost 40 years ago [10], [11]. Subsequently, underpowered linkage and candidate gene association studies determined a slow progress in the discovery of new genetic risk factors. In the past five years, genome wide association scans (GWAS) including thousands of patients and controls have allowed an unprecedented increase in the list of predisposition factors described for MS [12], [13]. Nonetheless, the individual effect of each variant is modest, with odds ratios ranging from 1.1 to 1.3. Moreover, the overall results of this statistically powerful approach account only partially for the MS heritability previously estimated by epidemiological studies. Approximately 30% of the genetic variation associated with MS is directly explained by variants represented by current GWAS arrays [14]. However, one should not forget that repetitive sequences of the genome skip the scanning of GWAS. Considering the results of the recently reported ENCODE project pointing to a pervasive transcription of the whole genome [15], these repetitive sequences could be potentially important to uncover part of the heritability not yet ascribed in these complex diseases.

An 8% of the repetitive sequences in the genome correspond to the so called human endogenous retroviruses (HERVs). They derived from the exogenous retroviral infection of the germline at different time points during evolution of the human lineage, and have been transmitted in a Mendelian fashion [16]. HERVs can be divided into distinct families, most of which resulted in multiple integrations of phylogenetically related but structurally heterogeneous elements. Host-retrovirus interactions influence the genomic landscape and have contributed substantially to mammalian genome evolution [17]. Some of the HERV familieshave been proposed to contribute to MS pathogenesis [18]. One of these HERV insertions associated with MS risk is HERV-Fc1, integrated about 10–15 million years ago [19]. Two subgroups exist in this family, HERV-Fc1 with only one component located in chromosome X and five related copies within the HERV-Fc2 subgroup. A polymorphism mapping close to the HERV-Fc1 copy located in chromosome X, rs391745, was found associated with MS in two out of the three Danish cohorts originally tested [20]. Then, further replication in a Norwegian cohort was achieved, revealinga specific association with bout-onset MS clinical forms [21]. Considering the mentioned effect of latitude on MS prevalence, we aimed to replicate the association of the HERV-Fc1 polymorphism rs391745 with relapsing remitting and secondary progressive MS in three independent Spanish cohorts, to ascertain whether the reported association is exclusive of higher latitudes in Europe or else is a general MS risk factor.

## Results

The first work providing genetic evidence for the involvement of HERV-Fc1 in the aetiology of MS and published by Nexo et al [20] studied the polymorphism rs391745 in three Danish cohorts: the discovery cohort showed a strong association [OR (95%CI) = 2.29 (1.60–3.28)], and from the two cohorts subsequently analyzed, one yielded replication [OR (95%CI) = 1.43 (1.09–1.89)] but the other did not evidenced association with MS [OR (95%CI) = 1.01 (0.64–1.59)]. Later, the effect observed in Danish MS samples was replicated in a Norwegian cohort [OR (95%CI) = 1.35 (1–1.83)] [21]. However, considering the accepted latitude gradient affecting MS prevalence, we aimed to validate this effect in a population from Southern Europe and to meta-analyze these results and those previously published (see Fig. S1 and Fig. S2).

A total of 2473 MS patients (see Table 1 for clinical data from the three cohorts) and 3031 ethnically matched Spanish controls were genotyped for rs391745 (Table 2). Genotyping success was over 95% for all groups of patients and controls. No departure from Hardy Weinberg equilibrium in the control groups was observed.

**Table 1:** Demographic characteristics of the MS patients included in the study.

	Northern Spain		Central Spain		Southern Spain	
*Characteristics*	Patients	Controls	Patients	Controls	Patients	Controls
Subjects (n)	569	980	883	692	1021	1359
Female (%)	73	46	66	55	68	69
HLA DRB1*15:01 (%)	42	25	38	11	38	18
Age (yrs) (Mean ± SD)	42±13	44±9	45±10	42±17	44±11	44±9
Age at onset (yrs) (Mean ± SD)	31±10	_	30±9	_	28±11	_
Clinical form:						
RR (n)	304	_	653	_	798	_
SP (n)	66	_	61	_	82	_
PP (n)	48	_	61	_	26	_

**Table 2:** Genotype frequencies of rs391745 in the Spanish cohorts included in the study.

		MS type	CC	CG	GG
			n (%)	n (%)	n (%)
**Northern Spain**	MS (n = 569)	Total	25 (4)	79 (14)	465 (82)
		15:01+	16 (7)	27 (11)	195 (82)
		15:01−	9 (3)	52 (16)	270 (81)
	Controls (n = 980)	Total	63 (6)	73 (7)	844 (86)
**Central Spain**	MS (n = 883)	Total	44 (5)	125 (14)	714 (81)
		15:01+	13 (4)	41 (13)	271 (83)
		15:01−	26 (5)	81 (15)	423 (80)
	Controls (n = 692)	Total	44 (6)	80 (12)	568 (82)
**Southern Spain**	MS (n = 1021)	Total	40 (4)	130 (13)	851 (83)
		15:01+	13 (6)	31 (13)	184 (81)
		15:01−	13 (4)	44 (12)	309 (84)
	Controls (n = 1359)	Total	71 (5)	208 (15)	1080 (80)

**Figure 1:**
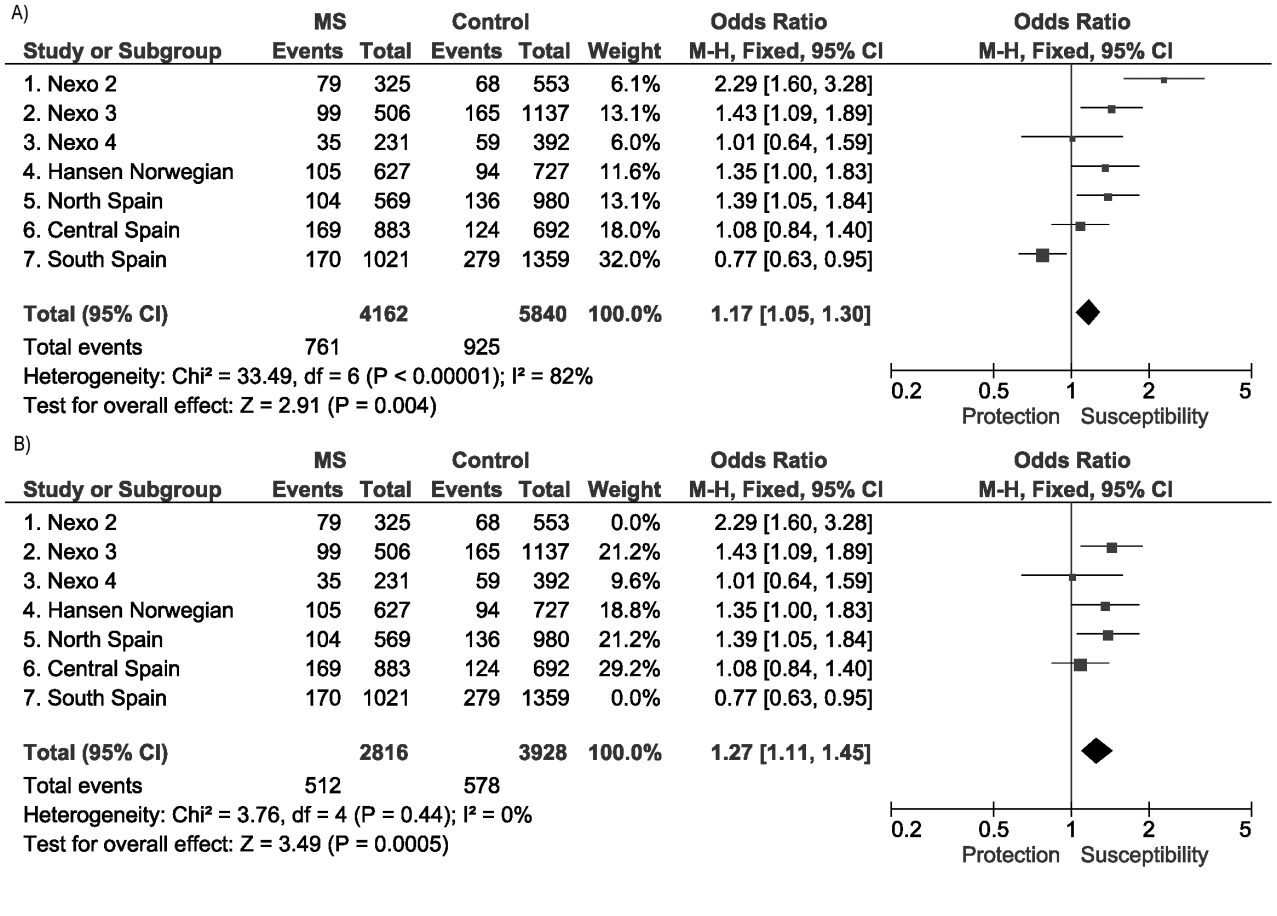
Meta-analysis including the studies of HERV-Fc1 association with MS published previously and those performed in the Spanish cohorts. Forest plot showing the overall data (A) and after eliminating heterogeneity by removing the Southern Spanish cohort (B).

As summarized in the forest plot depicted in Figure 1A, two out of the three tested Spanish cohorts showed significant associations of the rs391745*C-carriers with MS. However, in the cohort from Southern Spain an effect of the opposite allele could be detected. When heterogeneity was eliminated (I^2^ = 0%) by excluding this latter South-Spanish cohort and the discovery Danish cohort from Nexo et al [20], which was probably over estimated due to the so called *winner's curse*, the overall meta-analysisby Mantel-Haenszel test evidenced a strong association [Fig. 1B; p_M-H_ = 0.0005; OR_M-H_ (95%CI) = 1.27 (1.11–1.45)].

**Figure 2:**
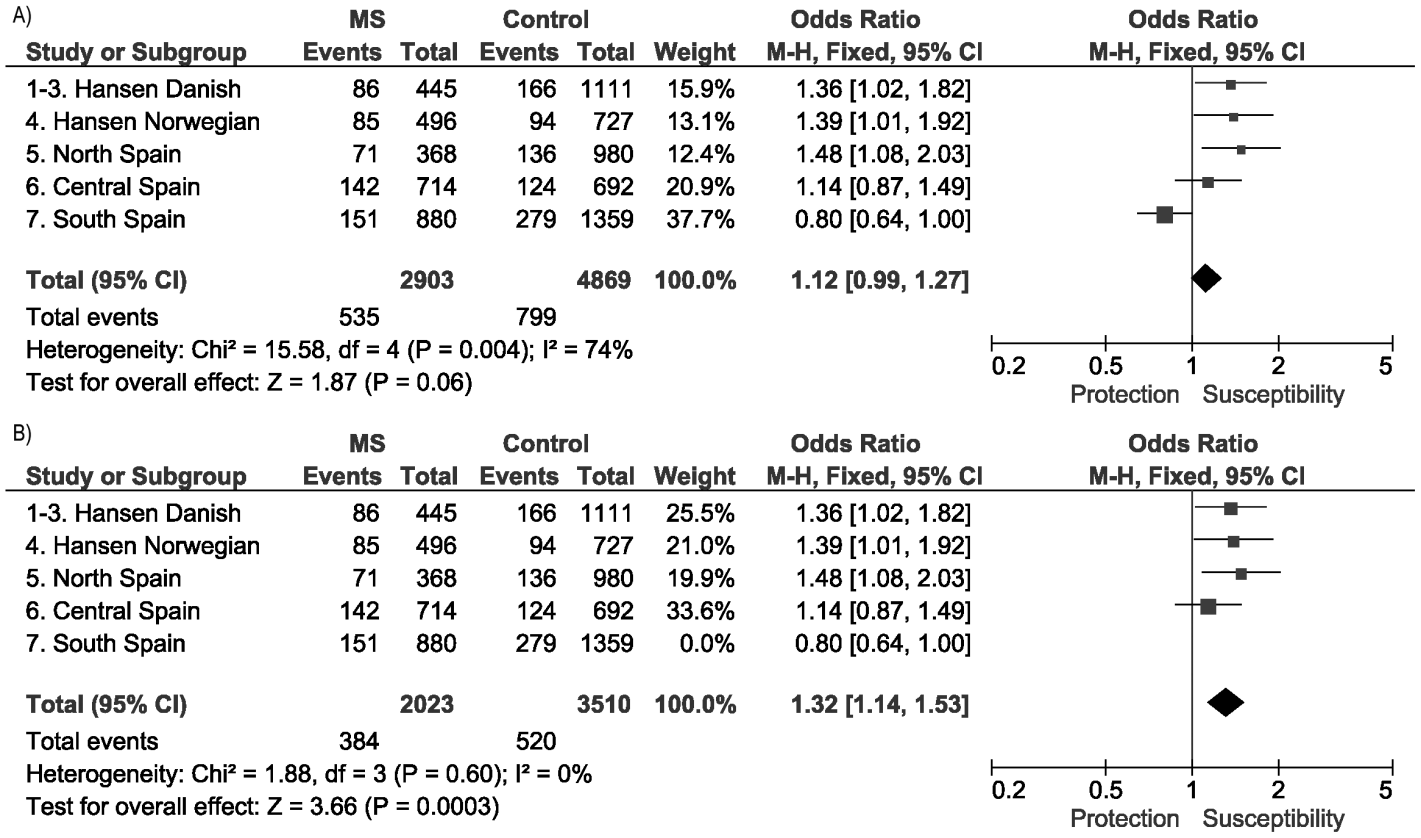
Meta-analysis of HERV-Fc1 association studies with bout-onset MS. Forest plot including all the available cohorts (A), and after eliminating heterogeneity by removing the Southern Spanish cohort (B).

Additionally, the study by Hansen et al [21] that included part of the already mentioned Danish cohorts, reported a specific effect of this polymorphism in bout-onset MS; in contrast, lack of association was claimed for the primary progressive clinical form. In the present work (Figure 2), when validation was restricted to relapsing remitting and secondary progressive MS samples from the Spanish cohorts that did not show heterogeneity in the previous overall analysis, a slight improvement in the association was observed [Fig. 2B; p_M-H_ = 0.0003; OR_M-H_ (95% CI) = 1.32 (1.14–1.53)]. Moreover, concordantly with the previous results, the pooled analysis of the primary progressive samples (n = 275) did not seem to follow a similar trend for association [OR_M-H_ (95% CI) = 0.92 (0.63–1.34)], although a definitive conclusion was hampered by statistical power limitations.

The stratified analysis by gender or HLA-DRB1*15:01 status did not detect any additional effect (Table 2).

## Discussion

The aim of this work was to validate the reported effect on MS susceptibility of a bi-allelic polymorphism located near the HERV-Fc1 insertion, rs391745, through genotyping of three independent Spanish cohorts. In the Northern European populations previously studied, this SNP located in chromosome X showed the most significant association amongst the 80 SNPs genotyped within a 20 Kb associated region which includes the endogenous retrovirus copy [20]. Environmental factors affecting MS have been recently reviewed [22] and the latitude gradient seems to be the more obvious, with a prevalence of MS oscillating more than 20 fold from very low levels near equator 5–10∶100000 to 200∶100000 in higher latitudes. In the present work, after a sensitivity analysis to remove the heterogeneous cohorts (I^2^ = 0%), a significant overall association was observed [p_M-H_ = 0.0005; OR_M-H_ (95% CI) = 1.27 (1.11–1.45)] suggestive of the role of the HERV-Fc1 insertion on MS predisposition.

The previously reported association was confirmed in cohorts from Northern and Central Spain, but a significant effect of the opposite allele was detected in the cohort from Southern Spain. This finding, already described in the literature as “flip-flop phenomenon” where significant associations for the same disease occur at opposite alleles of the same polymorphism, has been observed quite frequently [23]. Some authors hypothesized that this phenomenon can occur due to variation in linkage disequilibrium architecture [23], which is also present within the same ethnic origin [24]; while others explain it through differences in haplotypic frequencies [25]. Whatever the case, it has been proven that the probability of randomly observing a significant allele flip in samples ascertained similarly from a common population is negligible [26]. From this standpoint, the inclusion of the Southern Spanish cohort to the present meta-analysis considering association of the opposite allele would provide similar results [I^2^ = 0%; p_M-H_ = 0.0002; OR_M-H_ (95% CI) = 1.27 (1.12–1.45)].

**Figure 3:**
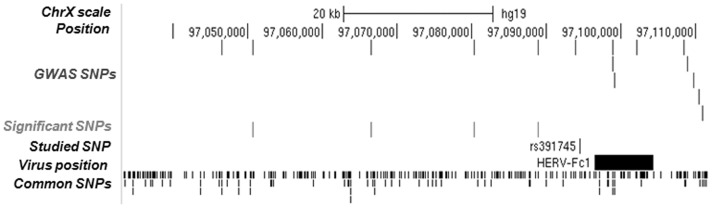
Scheme of the HERV-Fc1 position and polymorphisms in the locus. The figure depicts from top to bottom: Chr X scale; chromosomal position as indicated in B37/gh19 (Position); SNPs genotyped in the IMSGC-WTCCC2 GWAS (GWAS SNPs); statistically significant SNPs with p≤0.05 (Significant SNPs); position of rs391745 (Studied SNP); HERV-Fc1 retrovirus copy position (Virus Position) and common polymorphisms mapping at this locus (Common SNPs).

As Figure 3 summarizes, rs391745 was not genotyped in the last genome-wide association studies reported by the IMSGC-WTCCC2 consortium [13]. Nonetheless, four SNPs localized upstream to the HERV-Fc1 provirus showed association with MS even though they did not reach the GWAS threshold for significance (rs7881334, p = 0.002; rs402270, p = 0.005; rs318177, p = 0.0004; rs2125324, p = 0.016). These SNPs show high LD among them and their association with MS susceptibility is indicative of the relevance of this locus, although rs391745 shows low LD with these SNPs (r^2^<0.47). The Immunochip study, recently published in MS [27], was designed to deeply interrogate 184 non-MHC loci with genome-wide significant associations to at least one autoimmune disease, but none of them map to chromosome X where HERV-Fc1 is located, and therefore no additional information could be incorporated from this source.

In parallel to the genetic factors already described for MS [13], the effect now validated on MS predisposition is modest. This could explain why the size of the cohorts led to a limited statistical power to consistently detect the observed association and one of the Danish cohorts originally tested did not show association with HERV-Fc1 [OR (95%CI) = 1.01 (0.64–4.59)].

The proviral insertion of HERV-Fc1 in chromosome X presents stop-codons and a frame-shift in the *pol* gene; however, the *gag* and *env* genes are complete. A 4-fold increase in extracellular HERV-Fc1 gag RNA titres has been described in patients with active MS compared with healthy controls [28], but no additional insertions in other genomic contexts could be identified [29]. Further functional studies are warranted to elucidate the exact mechanism involved in the trigger of MS predisposition.

This new association transcends the boundaries of the identification of an additional genetic risk factor for MS, its location in a region of the genome overlooked by GWAS is highly relevant to dispatch the widely held view that a big part of the human genome constitutes mostly “junk DNA”, an issue recently illustrated by the ENCODE project [15]. The work now presented encourages the screening of unexplored sequences harbouring human endogenous retrovirus, as they could be at the crossroads of genetics and environment [30]. Some interesting results in terms of MS predisposition have been already published for HERV-W [31], HERV-H [32] and HERV-K18 [33], supporting the relevance of these endogenous retroviruses. A remaining challenge for the search of susceptibility variants in complex diseases as MS lies in populating these virgin territories by characterizing repetitive sequences of the genome.

## Materials and Methods

### Patients

Three independent cohorts including a total of 2473 MS patients and 3031 ethnically matched controls were consecutively recruited from the following Spanish Hospitals: H. Clínico San Carlos, Madrid (883 patients and 692 controls), H. Basurto, Bilbao (569 patients and 980 controls) and from three Hospitals of Andalucía: H. Regional Universitario, Málaga; H. Virgen Macarena, Sevilla and H. Virgen de las Nieves, Granada (1021 patients and 1359 controls) (Table 1). MS Spanish patients were diagnosed based on the McDonald criteria [34].

### Ethics statement

All subjects were recruited after written informed consent and the Ethics Committees of the participant hospitals approved this study: CEIC (Ethics Committee of Clinical Investigation) from H. Basurto (Bilbao), CEIC from H. Clínico San Carlos (Madrid), CEIC from H. Regional Universitario (Málaga), CEIC from H. Virgen Macarena (Sevilla), CEIC from C.S.I.C. and CEIC from H. Virgen de las Nieves (Granada). All research was conducted according to the principles expressed in the Declaration of Helsinki.

### Genotyping

DNA was extracted from peripheral blood by a standard salting out method. The single nucleotide polymorphism rs391745 was analyzed by Taqman technology using 384 well plates in a 7900HT Fast Real-Time PCR system, under the conditions recommended by the manufacturer (Applied Biosystems, Foster City, CA, USA).

### Systematic review

We performed a comprehensive search strategy of various electronic databases: MEDLINE (1966 - October 2013), Cochrane Database of Systematic Reviews (1991- October 2013) and EMBASE (1980- October 2013), by combining the terms: “HERV-Fc1”, “HERV-F” and “rs391745”. Additionally, a manual search of all references was conducted among the identified studies and relevant review articles. This search rendered 20 articles published to date. Neither date nor language restrictions were imposed. The association studies considered for further analysis were required to hold information about rs391745 genotypesand consequently only two studies remained. Non-published data from the Spanish cohorts described abovewere also included (see Fig. S1 and Fig. S2).

### Statistical analysis

Statistical analyses were performed with standard software (SPSS v15 and Review Manager RevMan v. 5.0.). For the Mantel-Haenszel analysis, Odds Ratios (ORs) and 95% confidence intervals (CIs) were calculated by using raw data for each study and for the pooled population. The Der Simonian and Laird random effects model was used according to the results of the tests of heterogeneity. The combined effect for heterogeneity was calculated by estimating the inverse variance, p value<0.10 and the I^2^ statistic with a cut-off point of 25%, which define a significant degree of heterogeneity between the studies. The effect of each study was weighted for the total number of patients included. A sensitivity analysis was performed to test the relative influence of each study on the results. Studies were sequentially dropped, and the effect on the change in the overall degree of heterogeneity was determined.

## Supporting Information

Figure S1 **Preferred reported items for systematic review and meta-analyses.** PRISMA 2009 Checklist.
(TIF)

Figure S2 **Preferred reported items for systematic review and meta-analyses.** PRISMA 2009 Flow Diagram.
(TIF)
