# Supplementary figures and images for: Human Endogenous Retrovirus HERV-Fc1 Association with Multiple Sclerosis Susceptibility: A Meta-Analysis
Source: PLoS One. 2014 Mar 3;9(3):e90182. doi: 10.1371/journal.pone.0090182 (PMC3971560; doi:10.1371/journal.pone.0090182)

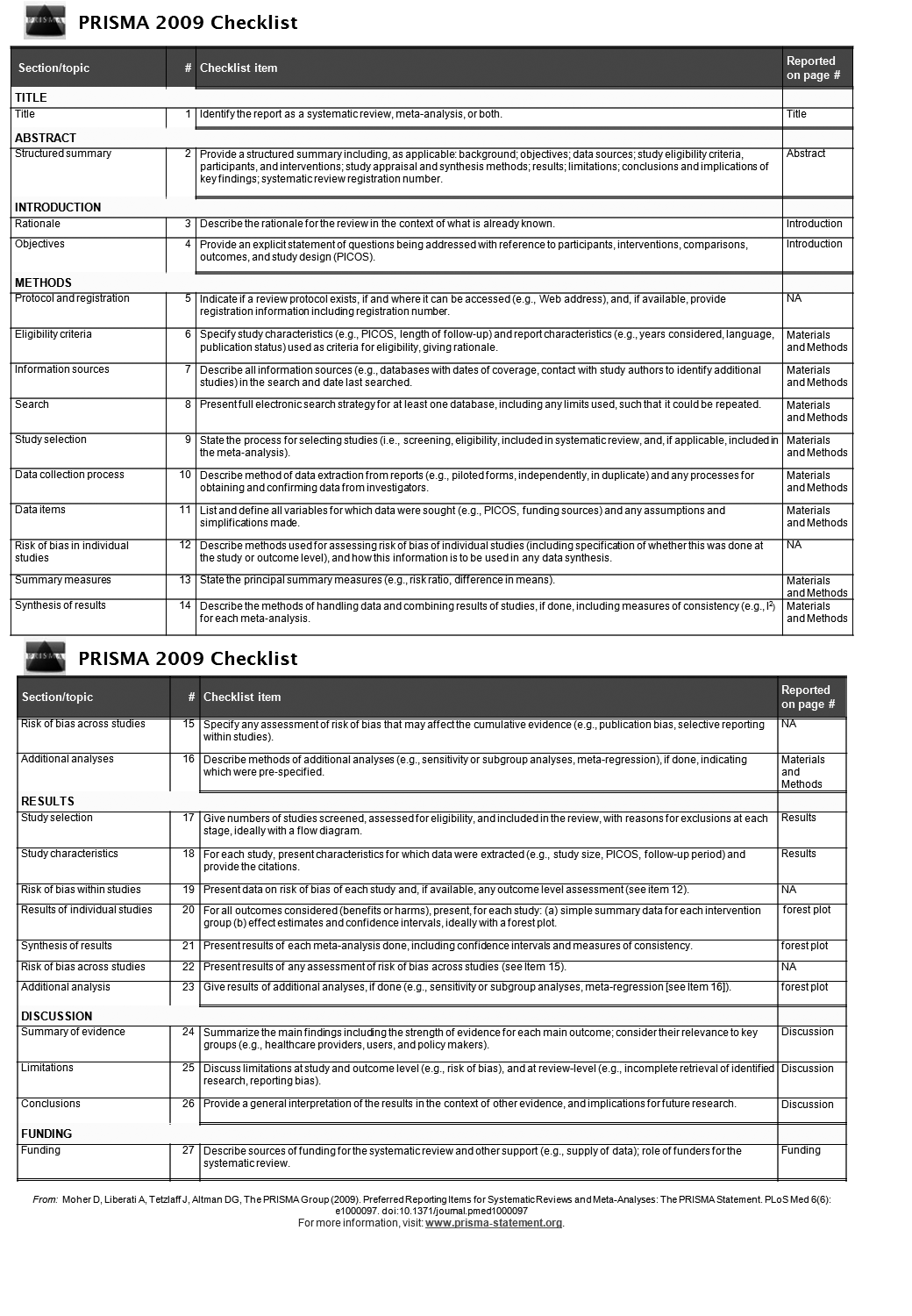

Supplement: Figure S1 — Preferred reported items for systematic review and meta-analyses. PRISMA 2009 Checklist. (TIF) [file pone.0090182.s001.tif]

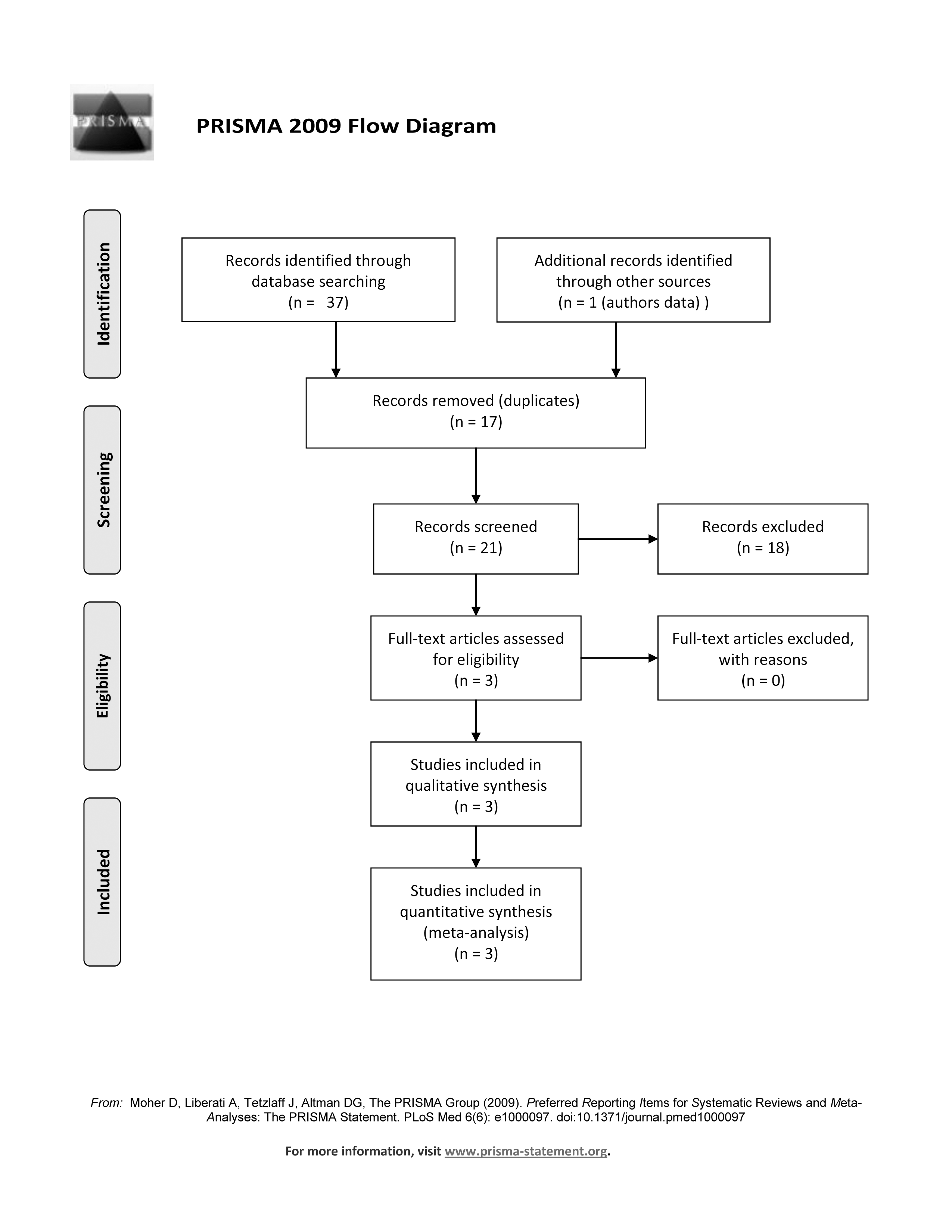

Supplement: Figure S2 — Preferred reported items for systematic review and meta-analyses. PRISMA 2009 Flow Diagram. (TIF) [file pone.0090182.s002.tif]
